# Pushing the Limits of Cancer Therapy: The Nutrient Game

**DOI:** 10.3389/fonc.2018.00148

**Published:** 2018-05-08

**Authors:** Daniele Lettieri-Barbato, Katia Aquilano

**Affiliations:** Department of Biology, University of Rome Tor Vergata, Rome, Italy

**Keywords:** mitochondria, diet, fasting, immunemodulation, microbiota and immunity

## Abstract

The standard cancer treatments include chemotherapy, radiotherapy, or their combination, which are generally associated with a multitude of side effects ranging from discomfort to the development of secondary tumors and severe toxicity to multiple systems including immune system. Mounting evidence has highlighted that the fine-tuning of nutrients may selectively sensitize cancer cells to conventional cancer therapies, while simultaneously protecting normal cells from their side effects. Nutrient modulation through diet also improves cancer immunesurveillance in a way that severe immunosuppression could be avoided or even the immune response or immune-based cancer therapies be potentiated also through patient microbiota remodeling. Here, we review recent advances in cancer therapy focusing on the effects of adjuvant dietary interventions (e.g., ketogenic diets, fasting) on the metabolic pathways within cancer cells and tumor environment (e.g., microbiota, immune system, tumor microenvironment) that are involved in cancer progression and resistance as well as cancer cell death. Finally, based on the overall literature data, we designed a nutritional intervention consisting in a plant-based moderate ketogenic diet that could be exploited for future preclinical research in cancer therapy.

## An Overview on the Control of Tumor Progression by Dietary Interventions

A plethora of epidemiological and experimental data demonstrated the efficacy of geroprotective dietary regimens (e.g., fasting, calorie, proteins, or single amino acids restrictions) in cancer prevention (1–3). Furthermore, such dietary patterns are emerging to be effective in selectively killing cancer cells, whereas increasing resistance of normal cells to the toxic effects of the anticancer therapeutics.

Calorie restriction (CR), defined as 30–60% less of daily calorie requirement without malnutrition, is known to extend healthy life span from yeast to mammals (4). The anticancer effects of CR are known since several years (5). CR is particularly effective in reducing the incidence, mass, and metastasis of breast cancer cells (6, 7). Remarkably, applying CR in combination with radiotherapy enhanced the radiotherapy efficacy inducing a more pronounced apoptosis of breast cancer cells than radiotherapy alone (7). In human, however, CR requires high compliance challenges to be maintained for adequate therapeutic period. For these reasons, short period of fasting without malnutrition have been proposed as potentially safe interventions to be associated with cancer treatments (8).

Fasting is commonly defined as a time-controlled deprivation of all kinds of foods and dietary nutrients. Differently to nocturnal fasting, time-controlled fasting leads to a profound metabolic reprogramming building up adaptive stress responses that are involved in life and health span extension (9–13). However, the adaptive stress responses induced by fasting occurring in normal cells differ from those activated by cancer cells because oncogenes might limit the activation of nutrient-sensing pathways while increasing chemotherapy vulnerability (8). Notably, proto-oncogenes such as IGF1R, PI3K, and AKT activate growth signaling and addict cancer cells to nutrient such as glucose and amino acids to meet their high proliferative rate (8). It has been shown that different cycles of fasting are effective in limiting tumor progression in several murine cancer models (14–17). However, the greatest effects were observed when fasting was combined with the conventional chemotherapy or radiotherapy (14–18). Interestingly, in these studies, fasting interventions alone do not cause clear signs of discomfort, but rather improve the animal condition. When fasting was combined with conventional therapies (e.g., temozolomide), most of the mice appeared healthy with the tumor-size below the controls, indicating that the combination of both treatments is well tolerated and improve tumor-bearing survival (14). The protective role of fasting against the side effects of anticancer therapy was confirmed in another study in which fasting was able to improve the overall cardiac response (maintenance of diastolic/systolic volumes and left ventricle wall thickness) to high-dose of doxorubicin (19). Fasting also exerted a significant protection against reduced mobility, ruffled hair, and hunched back posture caused by high dose of etoposide in mice (20). The anticancer effects of fasting might also rely on ketone bodies increase (21, 22). In support of this assumption, meta-analysis on ketogenic diets (KD), low in carbohydrates and high in fats, suggested a salutary impact on survival in animal models, with benefits prospectively linked to the magnitude of ketosis, time of diet initiation, and tumor location (23). Other evidence also demonstrated that KD might be safely used as adjuvant therapies to conventional radiation and chemotherapies (24). In particular, KD together with conventional radiotherapy led to increased radiation sensitivity in pancreatic cancer xenografts in mice (25). Similar results were obtained in mice bearing lung cancer xenografts (26). However, patients have demonstrated difficulty to comply with a KD while receiving concurrent radiation and chemotherapy in advanced lung and pancreatic cancer (25). Therefore, as better tolerated with respect to CR and KD, fasting appears to be more promising as adjuvant treatment in cancer therapy. Finally, it has been demonstrated that fasting could be replaced by the administration of CR mimetics, which showed the capability to improve the efficacy of chemotherapy as well. However, the objective response rates with metformin (27–30) or rapalogs (31) in clinical trials are still unclear and comparative analyses delineating a selective effectiveness of these drugs in cancer treatment and patient tolerability have to be more deeply elucidated.

## Nutrient Modulation in Proliferating/Resilient Cancer Cells: A Molecular View

The reduced levels of nutrients and growth factors observed during fasting led to hypothesize their mandatory role in governing the differential stress responses in normal and cancer cells (10, 14, 16, 18). The different responses of normal and cancer cells to fasting shed light on their different sensitivity to nutrients and growth factors (18).

**Figure 1:**
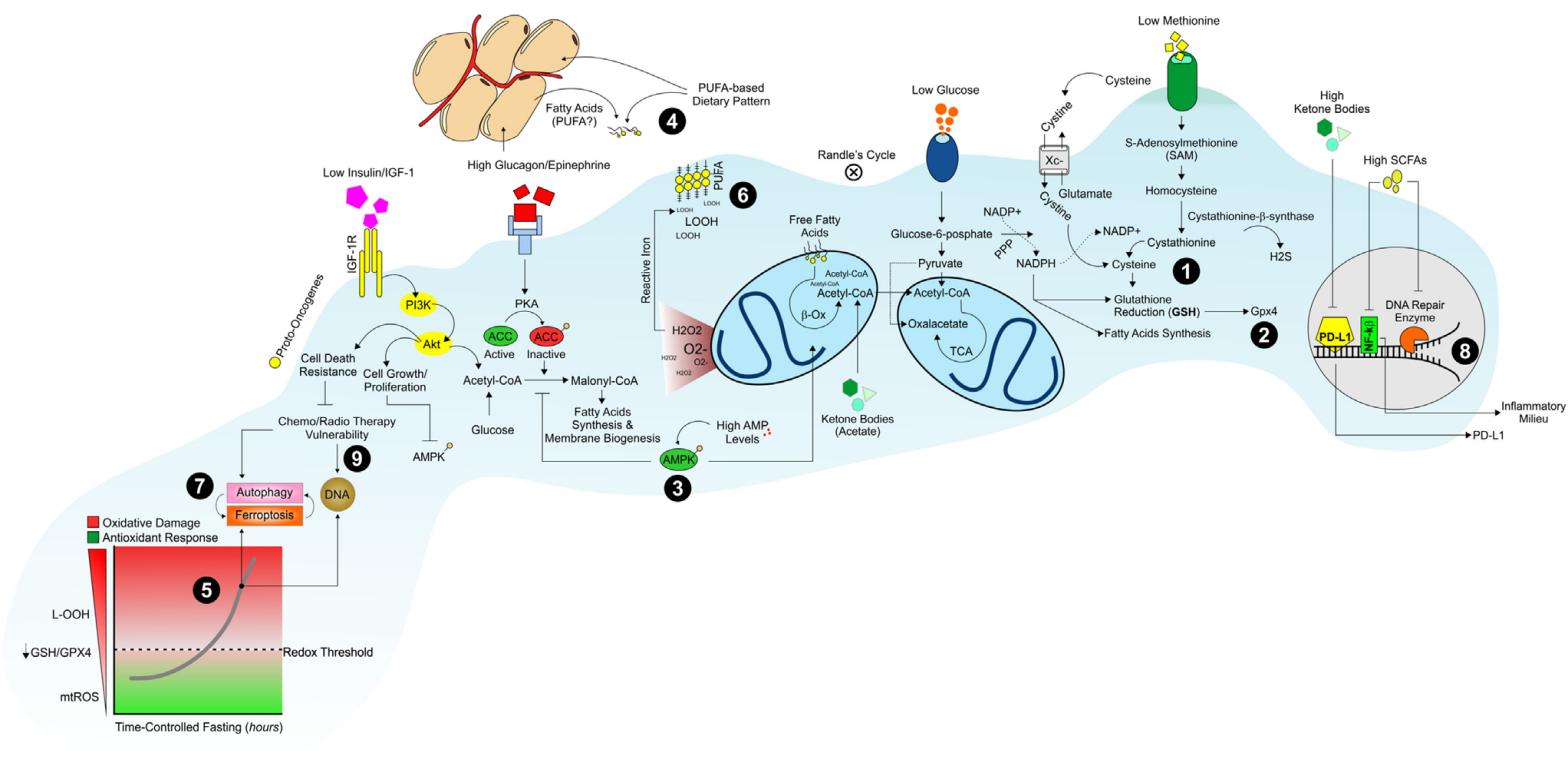
Dietary strategy to promote a hostile metabolism in proliferating/resilient cancer cells. Dietary patterns low in proteins, starch, and sugars promote an environment poor in sulfur amino acids (i.e., methionine, cysteine), glucose, and growth factors (insulin/IGF-1) that could limit NADPH/GSH production (1) and GPX4 activity (2). The diminished levels of glucose and glucagon/insulin ratio switch-off lipid synthesis and switch-on AMPK-driven lipid oxidation pathways in proliferating cancer cells (3). Under such metabolic conditions, cancer cells build-up their membranes by using extracellular dietary-derived and/or white adipose tissues-released fatty acids (4). The concomitant activation of OxPHOS metabolism and reduction in GSH levels are causative of oxidative stress (5) culminating in a massive lipid peroxidation (LOOH) (6) and ferroptosis in cancer cells (7). Diet low in starch, sugars, and proteins but rich in fatty acids also increases ketone bodies and modulates gut microbiota features by producing short-chain fatty acids (SCFAs). Ketone bodies and SCFAs affect PD-L1, nuclear factor-kb, and DNA repair enzymes genes transcription (8) promoting the chemo/radiotherapy vulnerability of cancer cells (9).

IGF-1/IGF-1R signaling is strongly dependent on nutrient availability and involves intensification of cancer cell proliferation, through the direct effects on PI3K/Akt signaling, and resistance to cell death imposed by chemotherapeutics and radiotherapy (Figure 1) (32). Indeed, fasting reduces circulating IGF-1 levels and this event protects mice deficient in the liver production of IGF-1 against chemotherapy drugs (16). Accordingly, restoration of IGF-1 was sufficient to reverse the protective effect of fasting (16). Reducing IGF-1 protects primary glia, but not glioma cells, against cyclophosphamide and mouse embryonic fibroblasts against doxorubicin (16). In the opposite manner, IGF-1 supplementation in starved breast cancer cells reversed drug sensitization. Overall, these findings strongly indicate that the fasting-mediated sensitization of cancer cells to chemotherapeutic drugs is conferred by the decrease of IGF-1 levels (15).

Nutrient shortage *per se* is able to increase mitochondrial reactive oxygen species (ROS) production in cancer cells arguing that limiting nutrient availability could enhance the effectiveness of redox-based cancer therapeutics (Figure 1) (33, 34). Actually, in breast cancer and melanoma cells, nutrient starvation was found to increase superoxide levels and aggravate oxidative stress caused by cyclophosphamide and cisplatin (15, 35). When applied in combination, fasting and chemotherapy act in synergy in elevating ROS levels and triggering DNA damage also in *in vivo* models of cancer (36). Micro-PET analyses in murine models of colon cancer cells revealed that fasting is effective as oxaliplatin (OXP) in reducing the average tumor glucose consumption and the lowest values were achieved by coupling fasting with OXP. In colon cancer cells, nutrient starvation upregulates oxidative phosphorylation with a significant production in mitochondrial superoxide caused by electron leakage. Consequently, starvation or OXP alone markedly increased ROS generation and their combination (starvation plus OXP) exacerbated ROS production in colon cancer cells (36). The hypothesis that cytotoxicity induced by glucose deprivation in cancer cells is mediated by mitochondrial superoxide and H_2_O_2_ was confirmed by exposing glucose-deprived transformed human fibroblasts to electron transport chain blockers (ETCBs), known to increase mitochondrial superoxide and H_2_O_2_ production (37). Glucose deprivation in the presence of ETCBs enhanced oxidative stress as well as cell death in several different human cancer cell lines (PC-3, DU145, MDA-MB231, and HT-29). In addition, human osteosarcoma cells lacking functional mitochondrial electron transport chain [rho(0)] were resistant to glucose deprivation-induced cytotoxicity and oxidative stress in the presence of antimycin A (complex III inhibitor), thus highlighting the role of mitochondrial ROS as mediators of cancer cell death (37).

The mechanisms by which KDs act as adjuvants in cancer therapy also seem to be associated with increased oxidative stress within cancer cells (24). Indeed, upon KD, the high level of circulating fatty acids limits the availability of glucose for glycolysis (Randle’s Cycle) (38). This reduces the formation of pyruvate and glucose-6-phosphate and in turn the synthesis of NADPH through the pentose phosphate pathway (PPP) (39). NADPH is necessary for buffering hydroperoxides (LOOH) production *via* the NADPH-dependent glutathione/glutathione peroxidase (GSH/GPX) system (40, 41). As consequence, an increase of LOOH is likely elicited (24) (Figure 1). Accordingly, hyperketotic diabetic patients have a higher level of lipid peroxidation in erythrocytes membrane and a significant decrease in cellular GSH levels than normal ketonic diabetic patients (42). Treatments with the ketone body acetoacetate elevated the levels of lipid peroxidation in human endothelial cells inhibiting their proliferation (42). This evidence suggests a direct role of ketone bodies in directly affecting GSH levels.

The main non-enzymatic cellular antioxidant GSH acts as an electron donor to reduce oxidized macromolecules, becoming itself oxidized in the process. Oxidized glutathione (GSSG) may then be restored in GSH through the action of the NADPH-dependent glutathione reductase (43). This enzymatic process generates NADP^+^, which may be reconverted to NADPH using electrons obtained from different biochemical pathways (44). Thus, proliferating cancer cells develop a peculiar metabolic flexibility to maintain a functional redox threshold by regulating NADPH levels through glycolytic flux modulation (33). Indeed, glucose-addicted human cancer cells cultured in a low-glucose medium without serum and amino acids are able to reprogram their metabolism by shifting toward PPP, which sustains the production of NADPH to dampen oxidative stress (33). However, during the initial stages of solid tumor development, when cells migrate to the lumen of lymphatic or blood vessels by loss of attachment (LOA) to the extracellular matrix, the glucose availability could not be sufficient to produce an adequate amount of NADPH and proliferation is inhibited (45). Upon such environmental stress, cancer cells induce adaptive responses consisting in the activation of AMPK signaling that inhibits fatty acid synthesis and triggers fatty acids oxidation to maintain energy production and NADPH levels (46, 47). Although cancer cells build up such adaptive responses, it has been observed that during LOA, cancer cells undergo ATP and NADPH drop and increase ROS production (48). Several papers demonstrated that cancer cells experiencing glucose shortage might maintain their proliferative capacity and membrane biogenesis by the uptake of extracellular lipids (49). Accordingly, extracellular saturated fatty acids supplementation supports the proliferative demand for biomass synthesis of proliferating cells (50, 51). Otherwise, supplementation with polyunsaturated fatty acids (PUFA) induced a significant cytotoxic effect on cancer cells either alone (52–54) or in combination with conventional anticancer therapies (55, 56). Differently to saturated fatty acids, PUFA are strongly susceptible to peroxidation (lipid peroxidation) in *in vivo* systems (57, 58). This appears to be a key mechanism triggering cancer cell death (59). With all this in mind, forcing the changes in the membrane lipids composition by dietary/nutrient enrichment in PUFA might promote an intrinsic sensitivity toward lipid peroxidation (57, 58, 60) and cancer cell death (Figure 1).

## Nutrient-Mediated Commitment to Ferroptosis in Cancer Cells

By preserving NADPH levels, cancer cells sustain GPX/GSH activity during nutrient limitation, and this may confer resistance to redox-based chemotherapeutics (61–63). Indeed, many rebel cancer cells use a common trick to evade annihilation; they enter into what is known as a mesenchymal state that is “epithelial-to-mesenchymal” transition, which provides cancer cell resistance to conventional therapeutic regimens (64). It has been demonstrated that high therapy-resistant mesenchymal cancer cells strictly rely on the selenium-dependent GPX4 for survival (65). By using the reducing power of GSH, GPX4 converts potentially toxic L-OOH to non-toxic lipid alcohols (L-OH) (Figure 1) (66–68). Accordingly, inactivation of GPX4 through GSH depletion with erastin, or with a direct GPX4 inhibitor, ultimately results in lipid peroxidation in cancer cells (69). It is thus provocative to hypothesize that the evolutionary pressure to maintain the selenium protein GPX4 might correlate with an organism’s requirement for an increased PUFA content, which, in turn, renders complex biological activities possible (70).

Uncontrolled lipid peroxidation is causative of the onset of a metabolically regulated cell death called “ferroptosis,” which is characterized by the iron-dependent formation of LOOH leading to cell death (Figure 1) (71). Sulfur amino acids play a key role in ferroptosis. In particular, agents that inhibit cystine uptake *via* the cystine/glutamate antiporter (XC system), such as sulfasalazine or erastin, arrest tumor growth and induce ferroptosis (72, 73). The uptake of cystine is followed by its NADPH-dependent conversion in cysteine, the rate-limiting amino acid precursor for the GSH biosynthesis (74). Direct depletion of cystine from plasma using an engineered cystine-degrading enzyme conjugate arrests tumor growth and triggers cell death (75). Agents that conjugate to GSH, as well as chemical or genetic inhibition of GSH biosynthesis, disrupt tumor cell growth and induce a ferroptosis-like form of cell death (76). Ferroptosis appears to be an effective cell death mechanism in cancer cells, since lipophilic antioxidant α-tocopherol or iron chelators, such as deferoxamine, efficiently dampen it (77). Hence, the presence of extracellular cysteine and cystine are crucial for growth and proliferation of various types of cancer, as these amino acids maintain GSH levels and prevent oxidative stress (Figure 1) (78–80). Because cysteine is limiting in the biosynthesis of GSH, some cancer cells, under cysteine unavailability, make use of the transsulfuration pathway to biosynthesize cysteine from methionine (Met), a dietary essential sulfur amino acid (81, 82). The essentiality of Met in cancer is supported by the evidence that some cancer cells display a higher sensitivity to Met shortage with respect to normal cells (83–87). The first steps of the transsulfuration pathway are the conversion to *S*-adenosylmethionine (SAM) and transfer of the methyl group of SAM to a large variety of methyl acceptors with formation of *S*-adenosylhomocysteine (SAH) (88), which can be then converted to homocysteine (Hcy) by SAH hydrolase (AHCY) (89). Alternatively, Hcy is converted to cystathionine by cystathionine β-synthase (CBS). CBS catalyzes the condensation of Hcy and serine, thereby forming cystathionine, which is subsequently cleaved to cysteine. Furthermore, exogenous cysteine is also essential for several cancer types (glioma, prostate, and pancreatic), as blocking its uptake through the cystine/glutamate antiporter reduces viability due to the cell death caused by uncontrolled oxidative stress (90–92). Similarly, CBS blockage reduces cancer cell proliferation and attenuates growth of patient-derived colon cancer xenografts models (93). Although these findings suggest that fasting or selective nutrient modulation could trigger ferroptotic cell death in cancer cells, a clear evidence linking nutrient availability to ferroptosis is still lacking. Several works demonstrated that starved cancer cells (mainly in amino acids) as well as cells lacking the enzyme producing NADPH from glucose (glucose-6-phosphate dehydrogenase) experience massive ROS production and autophagy-dependent cell death (33, 94, 95). Autophagy is a process described as intracellular removal of damaged organelles by self-degradative process (96). Interestingly, a tight relationship between autophagic cell death and ferroptosis is emerging (97–99). Indeed, it seems that autophagy activation leads to a degradation of ferritin (ferritinophagy) (97), thus increasing the intracellular free iron levels promoting ROS production and ferroptosis (Figure 1) (99).

## Dietary Strategies to Boost the Immunometabolic Responses in Cancer Therapy

Short-term fasting has a beneficial impact on cancer immunosurveillance (100). In particular, Pietrocola and co-workers demonstrated that fasting or CR-mimicking drugs, induce the depletion of regulatory T cells (which dampen anticancer immunity), thus igniting autophagic flux in murine models of KRAS-induced lung cancers. Accordingly, the inhibitory effect of fasting on tumor growth is lost in cancers that have been rendered autophagy deficient (100). Recently, also, isocaloric diet with protein restriction has been demonstrated to induce an IRE1α-dependent UPR in cancer cells, enhancing cytotoxic CD8^+^ T cell (a type of effector T lymphocyte)-mediated response against tumors (101).

**Figure 2:**
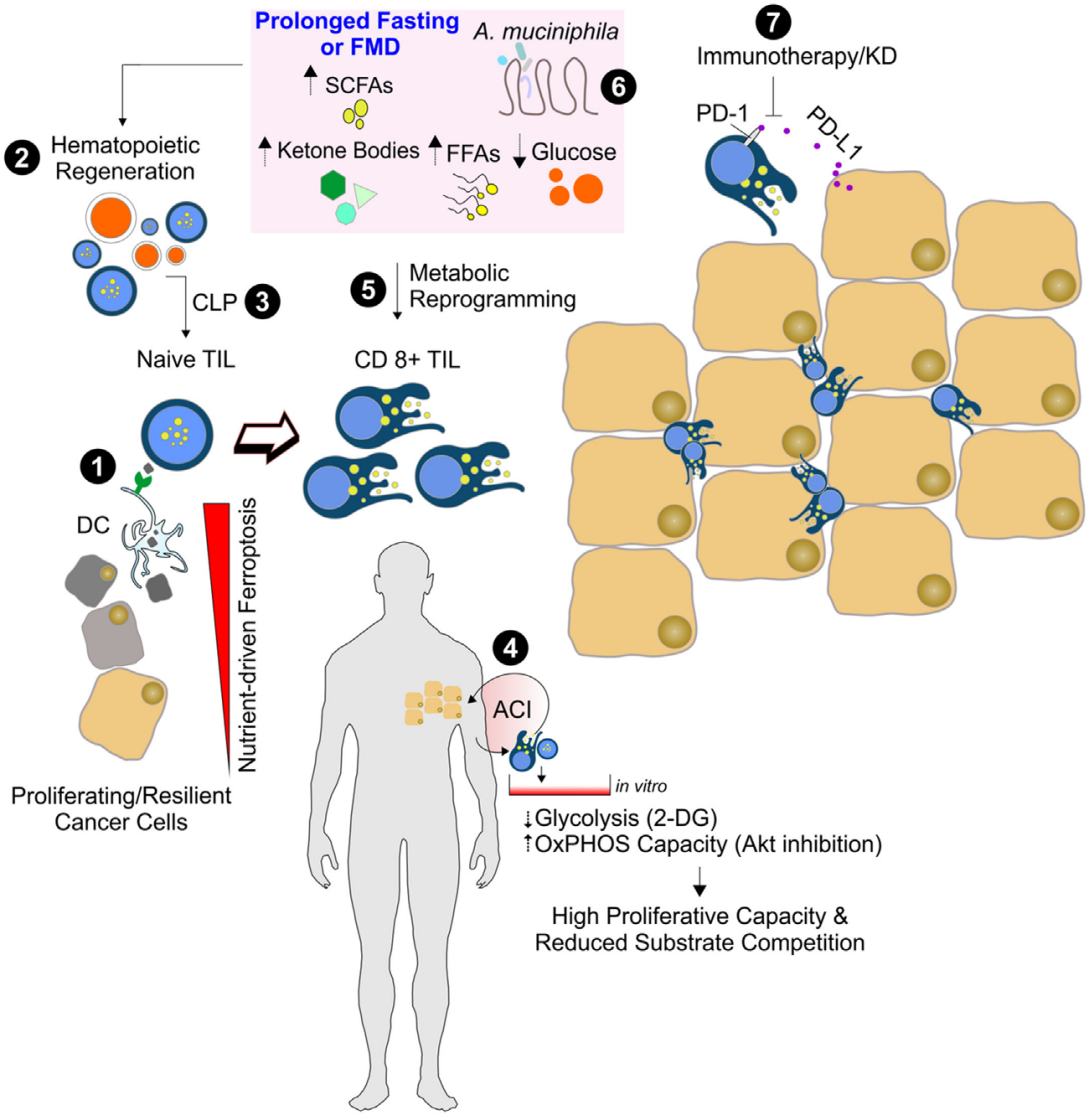
Nutrient manipulation to boost immunometabolic phenotype of CD8^+^ tumor infiltrating lymphocytes (TILs). Naïve CD8^+^ T cells recognize the antigen of ferroptotic cancer cells on class I MHC on dendritic cells, thus becoming mature cytotoxic CD8^+^ T cells (1). After prolonged fasting or fasting-mimicking diet (FMD), an enhanced hematopoietic regeneration rate (2) and enrichment of common lymphoid progenitor cells (CLP) can occur (3). The *in vitro* adoptive T cells immunotherapy (ACI) (4) and *in vivo* nutrient changes (5) reset CD8^+^ TIL metabolism toward mitochondrial oxidative pathways, thus limiting substrate competition with cancer cells and enhancing CD8^+^ TIL-mediated immunosurveillance. Dietary strategies promoting functional gut microbiota changes (e.g., *Akkermansia muciniphila* enrichment) (6) might improve the immune-checkpoint inhibitors (anti PD1/PD-L1) efficacy (7).

Similarly to what observed with prolonged fasting (102), cycles of a fasting-mimicking diet (FMD) are effective in increasing hematopoietic cells proliferation and promoting immune system regeneration and modulation (103). Importantly, FMD has stimulatory effect on common lymphoid progenitor cells and CD8^+^ T cell-dependent cytotoxicity on breast cancer and melanoma cells (Figure 2) (17, 102). The presence of cytotoxic CD8^+^ T cells in the tumor environment [tumor infiltrating lymphocytes (TIL)] is considered a positive outcome of the cancer treatment (104, 105).

CD8^+^ T cells are influenced by nutrients and other supportive signals that are generally available in their environment. Generally, tumor cells inactivate CD8^+^ T cells. The suppression of oxidative phosphorylation and an upregulated glycolytic flux of proliferating cancer cells create an immunosuppressive microenvironment (106). Indeed, the glucose-dependent CD8^+^ TIL could undergo a competitive disadvantage for nutrients, and this would negatively affect their immune function. The immunosuppressive metabolic environment could be further enhanced by tumor expression of inhibitory ligands for programmed death 1 receptor (PD-1) which, when bound to their cognate receptors on T cells, limits T cell-intrinsic glucose uptake and glycolysis (107, 108). It has been reported that KD significantly reduces the expression of the inhibitory ligand PD-1 (PD-L1) on CD8^+^ TIL (109). Additionally, mice fed with KD have reduced expression of PD-L1 on the cancer cells that notoriously inhibits CD8^+^ T cells activity (109). This suggests that KD may alter tumor-mediated T cell suppression by reducing the number of cells that are susceptible to inhibition through the PD-1 inhibitory pathway (Figure 2).

Nowadays, there has been intense interest in developing adoptive T cells immunotherapy (ACI), which consists in reintroducing into a patient T cells that are previously activated and expanded *in vitro* (110, 111). The success of the ACI depends on the replicative capacity of implanted T cells. A large amount of research has been directed in optimizing T cell activation and using appropriate adjuvants for ACI. However, few experimental studies have been focused on manipulating metabolic pathways that could potentially enhance immunotherapy efficacy. When posed in culture, T cells dispose of a high glucose availability, which is far from the glucose physiological levels especially in the tumor environment (112, 113). Thus, once reintroduced in patients, T cells suffer from low glucose levels and show a moderate survival and replicative capacity. It has been reported that limiting glycolysis in cultured T cells can increase their longevity without inhibiting proliferative capacity (114, 115) (Figure 2). Another potential way to enhance the replicative capacity and longevity of ACI cells is promoting oxidative phosphorylation and mitochondrial biogenesis *via* the inhibition of glucose-related signaling pathway that ultimately leads to *in vivo* persistence and improved antitumor immunity (116). The metabolic reprogramming of infiltrating glycolytic lymphocytes toward a catabolic state reliant on fatty acid oxidation appears to assure the success of immunotherapy (113). In line with this assumption, it was recently demonstrated that the enhancement of lipid catabolism in CD8^+^ T cells increases the efficacy of immunotherapy within a tumor microenvironment low in glucose (117). In a mouse model of malignant glioma, an enhanced cytolysis *via* tumor-reactive CD8^+^ T cells was also achieved by ketogenic diet (109). The immunometabolic reprogramming necessary for CD8^+^ TIL could at least partially explain the mechanism by which KD or fasting enhances cytotoxic effect against cancer cells. Such diets are indeed powerful in inducing a cellular metabolic shift from glycolysis toward FAO.

It is now emerging that CD8^+^ TIL response to immune checkpoint blockade inhibitor PD1 can be also modulated by gut microbiota (118–120). A very recent paper has revealed that fecal microbiota from patients affected with metastatic melanoma and responsive to anti-PD1 therapy display increased abundance of *Akkermansia muciniphila*. *A. muciniphila* introduction into mice receiving human nonresponder fecal microbiota transplant improved antitumor immune CD8^+^ T cell infiltration and activity and increased anti-PD1 therapy efficacy (120, 121). Another intriguing observation is that *Faecalibacterium* and *Bifidobacterium* are associated with anti-inflammatory responses, a regulatory arm of the immune system that aims to prevent overactivation of the immune response and restores host homeostasis (120). Given that changes in host metabolism and microbiota can occur in tandem, it was hypothesized that gut microbial diversity and composition are predictors of the response to cancer therapy (121) (Figure 2). Accordingly, germ-free mice implanted with human tumor cells and transplanted with feces from chemotherapy responders showed an ameliorated response to chemotherapy than mice colonized with microbiota from nonresponder patients (119).

The diet has a strong capacity to rapidly and reproducibly reshape the gut microbiome (122). Indeed, fasting or plant-based diet remodels microbial community structure and overwhelms interindividual differences in microbial gene expression. The animal-based diets are known to increase the abundance of bile-tolerant microorganisms (*Alistipes, Bilophila*, and *Bacteroides*) and decrease the levels of the high fermentative *Firmicutes* that metabolize dietary plant polysaccharides (*Roseburia, Eubacterium rectale*, and *Ruminococcus bromii*) (122). More recently, it has been demonstrated that alternate day fasting shifts the gut microbiota composition from *Bacteroides* to *Firmicutes* leading to elevation of the fermentation products (123). Plant-based foods are mainly characterized by resistant starches and dietary fibers and promote their gut microbiota-mediated fermentation and decomposition. These processes provide additional amount of short chain fatty acids (SCFAs) to the host (124) (Figure 2). The major SCFAs, i.e., acetate, propionate, and butyrate, have different production ratios and physiological activities. Through ^1^H NMR-based metabolomics, it was revealed that mice treated with alternate day fasting increased acetate levels both in the cecum and sera (123). Acetate, when ligated to coenzyme A (acetyl-CoA), is among the most central and dynamic metabolites in intermediary metabolism. Under stressful circumstance (e.g., fasting-like conditions), cancer cells may convert extracellular acetate to acetyl-CoA, thus promoting the biogenesis of membrane building blocks that sustain the high proliferative rate. This adaptive response involves the cytosolic form of short-chain acyl-CoA synthetases (ACC2). Accordingly, increased ACC2 protein levels were detected in a subset of human triple negative breast cancer samples, and such an elevation correlates with poor survival (125). Differently to acetate, butyrate shows many regulatory properties including the inhibition of histone deacetylases. Histone deacetylase inhibitors (HDACi’s) are emerging as promising anticancer drugs when administered alone or in combination with chemotherapeutic agents or radiotherapy. Previous research suggests that HDACi’s have a high degree of selectivity for killing cancer cells. For instance, the HDACi sodium butyrate suppresses DNA double strand break repair induced by etoposide more efficiently in MCF-7 cells than in HEK293 cells (126). Sodium butyrate alone also resulted in accumulation of ROS, DNA double-strand breaks, and apoptosis in HCT-116 colon cancer cell lines; when combined with mitomycin C or radiotherapy, sodium butyrate increases sensitivity of cancer cells to the drug (127, 128). In animal models of gastric carcinoma, sodium butyrate was found to inhibit tumor mass formation and increase tumor infiltration by CD8^+^ TIL (129). Finally, several studies also demonstrated a strong effectiveness of SCFA to inactivate nuclear factor-kb by downregulating the production of the pro-inflammatory cytokine TNFα (130–134), which is commonly activated to promote a pro-carcinogenic environmental milieu (135) (Figure 1).

## Conclusion and Perspective

Despite recent advances have been made in cancer therapy, the prognosis for many cancer patients remains poor, and current treatments still show severe adverse events. Thus, finding complementary treatments that have limited patient toxicity and simultaneously enhance therapy responses in cancer versus normal cells is urgent. Diet has a strong capacity to modulate cell responses to environmental stimuli and shows great potential in improving cancer prognosis. The mechanisms by which dietary nutrients enhance anticancer effects of standard anticancer therapies (chemotherapy, radiotherapy, immunotherapy) has not been fully elucidated yet. Preclinical studies have demonstrated the safety and efficacy of specific dietary interventions in counteracting tumor progression during anticancer therapy in murine models. However, most of the data present in the literature take advantage of the use of mice and this may limit the translation to clinical research. Therefore, a huge amount of work is now necessary to confirm these very promising results in humans.

**Figure 3:**
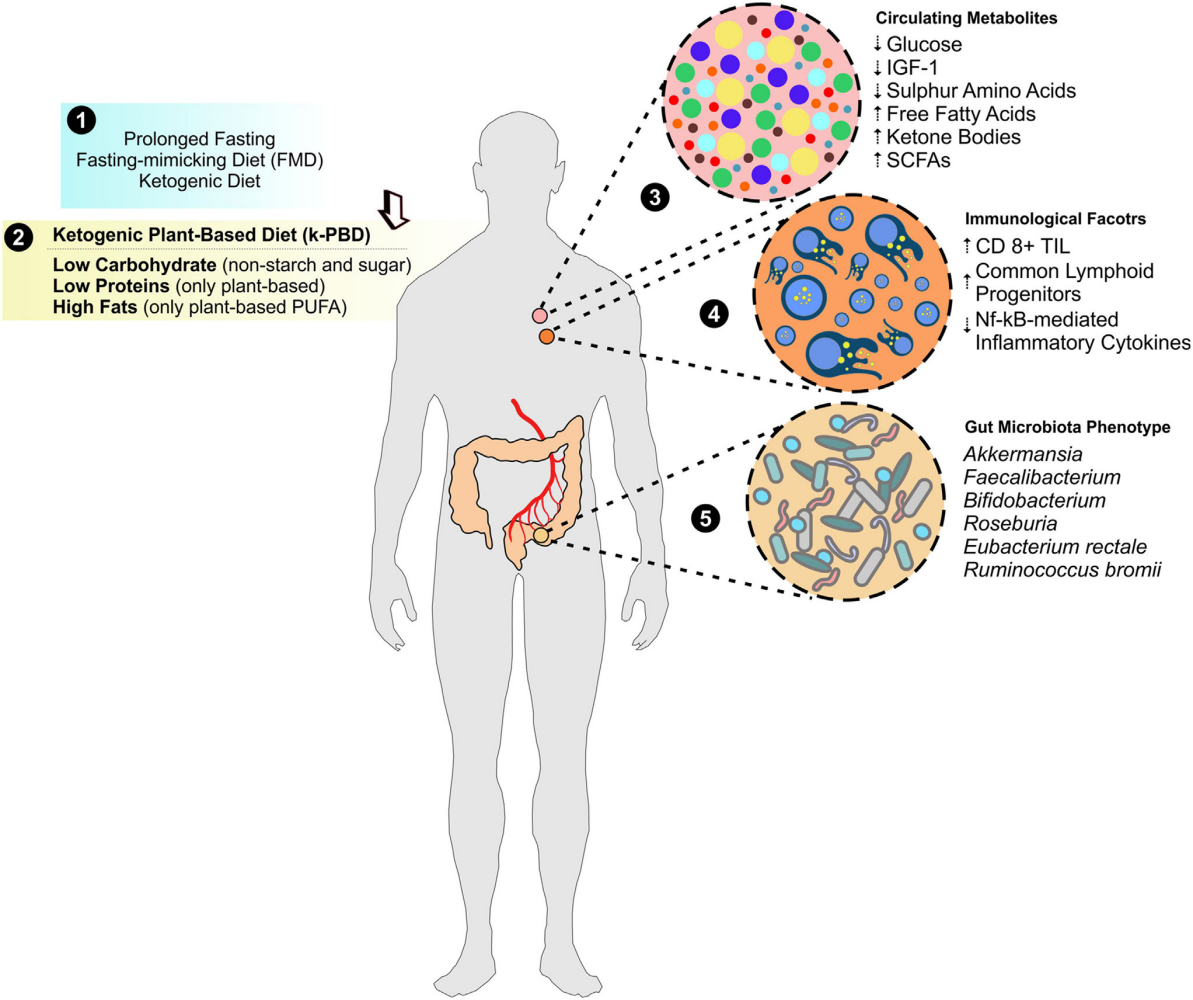
Improving metabolites and immunological anticancer profile by k-PBD. Evidence from prolonged fasting, fasting-mimicking diet (FMD), and ketogenic diet demonstrated a strong usefulness as adjuvants in cancer therapy (1). In this issue, we propose a moderate ketogenic plant-based diet (k-PBD), low in carbohydrates (starch and sugars in particular) and animal proteins (poor in sulfur amino acids and selenium) but rich in fats [mainly in vegetable polyunsaturated fatty acids (PUFA)] (2), which could strongly modulate circulating metabolites (3), immunological factors (4), and gut microbiota asset (5) that overall create a hostile environment to cancer cells.

Deprivation of nutrients (e.g., glucose, sulfur amino acids) as well as of nutrient-responsive growth factors (e.g., IGF-1) seems to selectively kill high proliferative/resilient cancer cells by forcing their glycolytic asset toward an oxidative metabolism (i.e., fatty acids and ketone bodies as energy sources) and limiting GPX activity as consequence of reduced GSH levels. Nutrient scarcity also improves immunometabolism enhancing cytotoxic efficiency of CD8^+^ TIL within the tumor mass through, probably, the concomitant gut microbiota and immunometabolic rearrangements (Figure 3).

**Figure 4:**
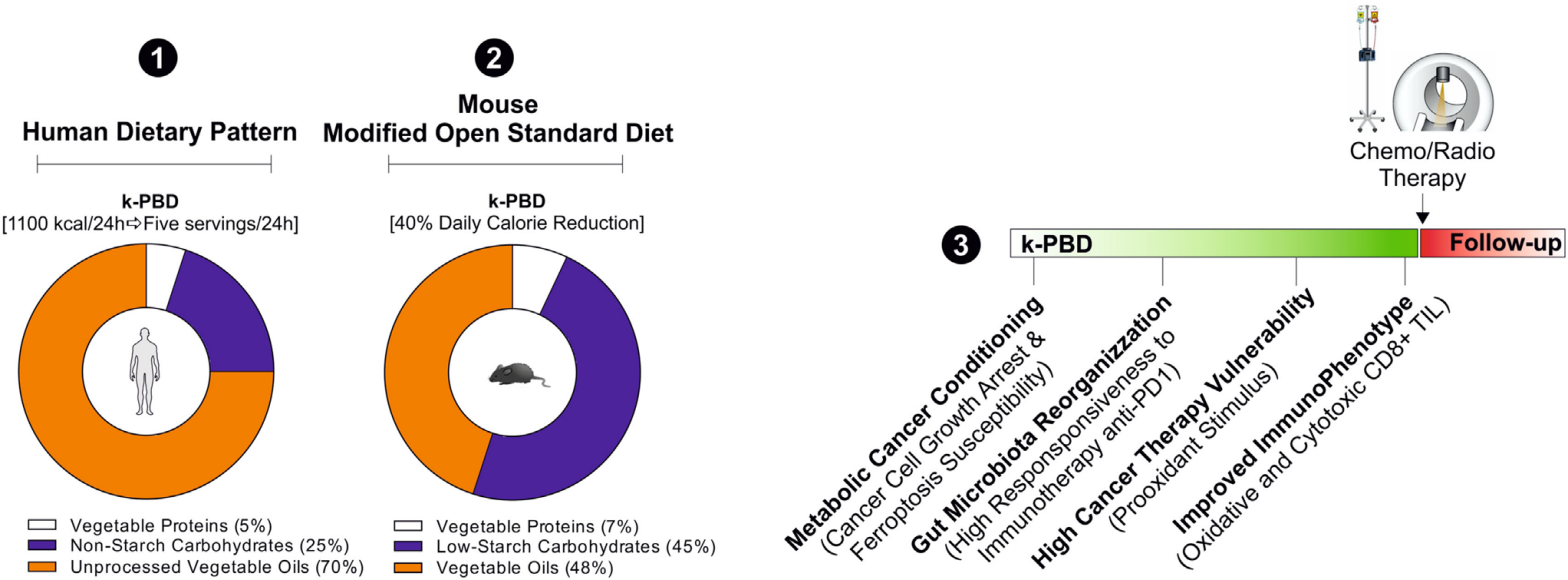
Hypothetical cancer phenotypic responses to k-PBD. For the human dietary intervention studies, k-PBD (for 4 consecutive days; 5 servings each day) should provide about 1,100 kcal/die with 5% of calories from vegetable proteins, 25% from carbohydrates (non-starchy vegetables), and 70% from unprocessed vegetable oils (1). k-PBD proposed for murine cancer models should be reduced in total calories (40% reduction versus *ad libitum* diet), provide 7% calories from vegetable proteins, 45% from low starch carbohydrates, 48% in vegetable oils (2). The proposed dietary pattern should be started prior to conventional cancer therapies (3).

Herein, we propose weekly cycles of 4 days of a plant-based moderate ketogenic diet (k-PBD) that could reprogram systemic metabolism conferring a hostile environment to cancer cells (Figure 3). In particular, k-PBD should be low in proteins (mainly vegetable proteins low in sulfur amino acids and selenium), carbohydrates (non-starchy vegetables), and high in lipids (mainly unprocessed vegetable oils rich in PUFA). Remarkably, even though not supported by experimental data, it is highly expected that this diet could be able to increase ketonemia as it contains high amounts of fats concomitantly to reduced calories. This diet could increase the efficiency of CD8^+^ TIL, by reprogramming their metabolism (fat-dependent metabolism) to better counteract the metabolic features of proliferating cancer cells (glucose-dependent metabolism) and sensitize cancer cells to the therapy. The k-PBD could be consumed prior to conventional cancer therapies (e.g., prior each cycle of chemotherapy or prior a single fraction of radiation therapy). With this composition and time of treatment, k-PBD could be effective in: (i) changing the membrane chemistry by PUFA enrichment (high peroxidation index); (ii) reducing the sulfur-dependent antioxidant power (lowering NADPH, GSH, GPX4); (iii) forcing the metabolic shift toward mitochondrial metabolism in cancer cells. Furthermore, the high fermentative fibers of k-PBD could induce a functional microbiota reshaping improving immunotherapy efficacy (e.g., anti-PD1 therapy) (Figure 4).

## Author Contributions

DL-B conceptualized and wrote the manuscript. KA performed critical revision of the manuscript for intellectual content.

## Conflict of Interest Statement

The authors declare that the research was conducted in the absence of any commercial or financial relationships that could be construed as a potential conflict of interest.
